# Economic Impact of Leading Prosperity Diseases: COPD in South East Europe

**DOI:** 10.3389/fpubh.2015.00050

**Published:** 2015-03-24

**Authors:** Vojislav Cupurdija

**Affiliations:** ^1^Faculty of Medical Sciences, University in Kragujevac, Kragujevac, Serbia; ^2^Clinical Center Kragujevac, Clinic for Pulmonary Diseases, Kragujevac, Serbia

**Keywords:** COPD, community acquired pneumonia, cost, resource use, South Eastern Europe

## Economic Burden of COPD

Chronic obstructive pulmonary disease is one of the leading “prosperity diseases” worldwide. Pooled global prevalence rates based on clinical assessments and spirometry ranged from 7.6 to 8.9%, reported in a sound meta-analytical study design (1). It has far reaching consequences, not only for an affected patient’s health but also for the entire national health systems (2). These refer to the substantial work load for the medical facilities due to chronic clinical course of illness and modest success of available treatment approaches. COPD attributable resource utilization patterns are particularly substantial if large university tertiary care hospitals, specialist clinics, and intensive care units are observed (3). According to most of published evidence the key cost driver are periodic exacerbations followed by intensive care unit admissions and episodes of infectious complications (4). Among major cost domains, physician consultations and surgery dominate in high-income settings. Unlike in the West, within the most of South Eastern European region, COPD medical care is still dominated with acquisition costs of pharmaceuticals and oxygen (5) and imaging diagnostics (6). Outpacing of indirect productivity-related opportunity costs by the direct costs of in- and outpatient medical care is common to this region due to substantially lower wages of physicians and nursing staff (7). Apart from direct costs of COPD, mainly constituted from the resources consumed in the health care process, including costs of ambulatory care, drug treatment, hospital care, rehabilitation, and long-term home care, there are substantial indirect costs of COPD, which are incurred by productivity losses, premature retirement, and premature mortality from this disease. The indirect costs for premature mortality are being calculated through human capital approach, with the life years lost up to the age of 65 multiplied by the gross annual income. An insight into the economic reality of SEE region, particularly Serbia, with average wages significantly lower than in countries of the Western Europe, but at the same time with high unemployment rates in younger age groups, where some 50% of the working population is currently outside of the workforce (8), being in their most productive decades of life but at the same time most prevalent tobacco users, makes indirect cost of COPD in SEE region very difficult to calculate or even predict, but clearly shows significant magnitude of this burden in present years, and probable rise of these costs in the future. Intangible costs are not convertible into monetary terms and units, they are specifically related to the distress and suffering, which is caused by the disease. General lack of insight into patients’ perception of the disease and limitation and incapability, which it imposes, while healthcare workers are being focused mainly on physical burden of the disease, with very few patients being provided with structurised psychosocial aid in the attempt to overcome significant yet underestimated mental and emotional burden of the disease, makes these costs impossible to predict and foresee.

## An Example of Serbia’s Health Reforms

Serbia as the largest Western Balkans upper-middle income market began health reforms one decade later than most transitional countries of SEE region (9, 10). After dynamic 2000–2007 GDP increase and overall development, the issue of long-term sustainability of its health system financing became hot topic under the first strike of global recession (11). Large part of almost unbearable economic burden was attributable to the major prosperity diseases including pulmonary diseases (12). The unique common weakness revealed by all of these pioneering cost-of-illness assessments in the Balkans region was poor health system responsiveness to population needs together with overextended hospital budgets and accumulating of public depth generated by the national health insurance fund (13). An occurrence of catastrophic household expenditure triggered by severe illness, sinking entire families into poverty is still prevalent within the society (14). Expensive medical technologies, which were denied reimbursement, remained mostly unaffordable to the ordinary citizens (15).

## Consequences of COPD Relative to Population Aging and Comorbid Disorders

Populations across Eastern Europe and the Balkans are aging even more rapidly than their Western European counterparts. This population aging is likely to further constrain already limited resource allocation in health care (16). This happens mostly due to dwindling base of employed tax payers in their most productive life time combined with increased proportion of the elderly. Demand for medical services by the retired citizens is significantly higher compared to working, younger age groups and this is particularly the case within the last year of life (17). Substantial impact of age to the COPD costs of care was already claimed in literature. Clinical severity of disease according to the Global initiative for chronic Obstructive Lung Disease (GOLD) classification, clearly correlated with resource use and costs of hospital and outpatient care (18).

In an exploration of long-term pharmaceutical market transformation trends in Western Balkans, it has been observed that agents used to treat COPD exhibited prominent rise in market share during the past decade. Reported value based turnover of medicines intended to treat respiratory disorders grew from € 17,090,000 in 2004 to the € 46,500,000 in 2012 (19). It is a paradox that during same 9 years unit drug consumption in terms of defined daily doses DDD/1000 inhabitants/day actually fell from 164.55 in 2004 to 50.55 level in 2012 [according to Anatomical Therapeutic Chemical (ATC) classification (“R” ground code group)] (20). Explanation for this shift in Balkan pharmaceutical markets should be looked within stronger brand penetration and modest success of generic pharmaceuticals in many therapeutic areas (21). COPD-related prescription and dispensing of β-adrenergic preparations in combination with inhalatory corticosteroids (“R03AK” ATC code group) were reported record breaking fivefold increase from € 2,682,320 in 2004 toward € 11,761,775 in 2012 based on latest official release by the National Medicines and Medicinal Device Agency of Serbia (22). Recent dissertation conducted on health economics of community acquired pneumonia (CAP), proved clear proof of substantial COPD comorbidity impact to the overall costs of medical care. While ordinary CAP clinical cases incurred on average € 717 costs in a 1 month time horizon while the ones suffering from COPD and CAP incurred € 970 monthly costs of inpatient care (23).

## Proposed Measures to Tackle the Challenges Lying Ahead

Although COPD prevalence and incidence seem to be steadily slowing down in some parts of the European region, this might not be the case with mortality rates. Unfortunately, COPD will most likely be the third leading cause of death worldwide and the fifth leading cause of years lost through early mortality or handicap (disability-adjusted life years) in 2020, which is far worse landscape compared to 1990 (24). So far serious policy initiatives to combat decreased longevity and quality of life caused by COPD have been taken both by WHO and the European COPD Coalition (ECC) (25). Orchestrated supranational efforts to increase research investment in the therapeutic options for COPD were proposed within the Horizon 2020 framework as well (26).

Broad forecasts on COPD for the South Eastern European region may be significantly less optimistic compared to the traditional EU15 economies (see Table 1). Some underlying reasons are strong popular affection toward smoking tobacco among adolescents (27, 28) and inefficiencies of anti-tobacco public health campaigns and policies (29). Environmental pollution plays less significant role due to shutting down of most heavy industries in former socialist countries (30) due to socioeconomic transition as well as their lack of global competitiveness (31). Popular opinion on tobacco is gradually beginning to change but this is likely to be a lengthy process. Additional obstacles to this tobacco reduction process seem to be traditionally high prevalence of tobacco smoking habit in Balkan countries such as Bulgaria (32) and FYR Macedonia (33) and heavy investment of global multinational tobacco manufacturers in Serbia and Turkey in particular (34). Essential revenues provided to the local governments by taxation of tobacco sales both to the industry and the consumers is still too important to the regional economies, still outside EU, such as Western Balkans and Turkey. This fact makes tobacco control policies currently in place less successful. Opposingly, promising trend of decreasing tobacco consumption is clearly visible in the OECD economies such as Greece (35), Slovenia, Hungary, and Cyprus where smoking free legislation, higher taxes on cigarettes, and facilitated access to medicines used to treat nicotine addiction are being applied for a number of years in line with the EU health priority targets (36). According to combined tobacco control score (TCS), most countries of South Eastern European region obtained <50 grades with the exceptions of Ukraine and Turkey. Interestingly, unsatisfactory and weak tobacco control policies remain in place in a number of traditional high-income European economies.

**Table 1:** **Ground indicators on respiratory disorders, tobacco consumption, and health expenditures in SEE 1980–2010**.

Country	AL	BA	BG	HR	CY	GR	HU	MN	MD	RO	RS	SK	SI	MK	TR	UA
SDR, bronchitis/emphysema/asthma, all ages, per 100,000 – 1980	26.08^1987^	36.77^1985^	41.91	27.86^1985^	12.89^2004^	20.64	62.15	1.7^2000^	20.1^1981^	71.71	28.67^1998^	31.97^1986^	14.74^1985^	40.25^1991^	N/A	26.57^1981^
SDR, bronchitis/emphysema/asthma, all ages, per 100,000 – 2010	13.07^2004^	15.33^2011^	10.45	21.06	9.31	0.17	31.1	0.12^2009^	42.3	20.5	23.04	13.09	12.2	18.74	35.83	31.16^2004^
SDR, selected smoking-related causes, per 100,000 – 1980	262.81^1987^	269.46^1985^	544.43	353.88^1985^	158.33^2004^	292.22	566.24	243.38^2000^	844.85^1996^	461.93	382.43^1998^	440.38^1992^	347.76^1985^	335.44^1991^	N/A	637.25^1991^
SDR, selected smoking-related causes, per 100,000 – 2010	324.09^2004^	237.58^2011^	345.25	349.9	128.09	183.07	425.16	180.62^2009^	762.36	427.69	332.47	416.46	185.02	331.21	232.04	774.79^2004^
Hospital discharges, respiratory system diseases, per 100,000 – 1980	1954.46^1989^	850.83	2743.52	1351.22^1981^	773.5	1193.82	1854.34^1992^	1678.87^1988^	3830.1	3089.9	812.19^2000^	1958.95^1991^	1721.4	970.65^1983^	329.85	4930.78
Hospital discharges, respiratory system diseases, per 100,000 – 2010	1331.93	855.92^1989^	3098.79	998.13	599.76^2008^	1536.89^2007^	1685.35	1275.22	2467.71	2817.71	1106.06	1471.01	1410.74^2009^	1863.55	1781.6	3704.3
Prevalence of chronic obstructive pulmonary disease (%) – 1980	0.15^1994^	3.14	2.6	0.41^1981^	N/A	0.35	0.12^1988^	N/A	1.3^1991^	0.49^1989^	N/A	1.02^1994^	N/A	0.17^1983^	N/A	2.76^1996^
Prevalence of chronic obstructive pulmonary disease (%) – 2010	0.21	1.56	2.25^2000^	0.14	N/A	0.24^2008^	1.47	N/A	N/A	1.47	N/A	1.68	N/A	0.38^2007^	N/A	3.94
Number of cases of chronic obstructive pulmonary disease – 1980	4870^1994^	128449	230335	18745^1981^	N/A	34117	37278^1990^	N/A	56878^1991^	113814^1989^	N/A	54545^1994^	N/A	3359^1983^	N/A	1403640^1996^
Number of cases of chronic obstructive pulmonary disease – 2010	6874	59968	184167^2000^	6200	N/A	26595^2008^	147480	N/A	N/A	315437	N/A	91023	N/A	7737^2007^	N/A	1799851
% Of regular daily smokers in the population, age 15+ – 1980	29.5^1990^	37.6^2002^	31.4^1986^	32.6^1995^	23.9^2003^	46^1991^	44^1992^	N/A	19^2000^	25.9^1989^	33^2000^	24.4^1992^	34^1988^	N/A	44^1988^	40^1990^
% Of regular daily smokers in the population, age 15+ – 2010	39	14.3	39.7^2007^	27.4^2003^	26.5^2008^	31.9^2009^	31.4^2009^	32.7^2008^	27.1^2006^	26.7^2011^	26.2^2006^	19.4^2009^	19.2^2012^	36^1999^	25.4	23.3
Number cigarettes consumed per person per year – 1980	436.19^1996^	820.82^1997^	1880.95	2167^1992^	N/A	2271.4	2652.39	N/A	N/A	1347.07^1991^	N/A	1715.05^1993^	2500.54^1996^	2143.11^1996^	1167.44	N/A
Number cigarettes consumed per person per year – 2010	744.06^2000^	1244.01^2000^	2792.6^2000^	1736.68^2000^	N/A	3200.49^2004^	2151.41^2000^	N/A	N/A	1392.63^1997^	N/A	1230.4^2000^	2232.86^2000^	1794.36^2000^	1547.84^1998^	1027^2000^
Sulfur dioxide emissions, kg per capita per year – 1980	N/A	107.19^1990^	231.34	32.72	N/A	41.48	152.46	N/A	76.78	47.52	N/A	156.49	123.41	N/A	4.59	77.14
Sulfur dioxide emissions, kg per capita per year – 2010	N/A	N/A	104.77^2000^	15.98^2000^	N/A	50.01^2000^	53.86^2000^	N/A	31.61^2000^	40.12^1994^	N/A	38.88^2000^	13.57^2000^	52.3^1998^	15.49^2000^	46.91^2000^
Average annual concentration of sulfur dioxide (SO_2_) in capital, μg/m^3^ – 1980	N/A	18.4^2002^	27.4^1998^	N/A	N/A	22.2^1997^	41.6^1997^	N/A	N/A	N/A	58.6^2003^	25.4^1997^	35.4^1997^	27.3^1997^	N/A	N/A
Average annual concentration of sulfur dioxide (SO_2_) in capital, μg/m^3^ – 2010	38.5^2009^	35.1	9.4	N/A	N/A	5.7^2008^	6.7	N/A	N/A	15	37.8	19.5	2^2008^	1.3	12.6	N/A
Average annual concentration of particulate matter <10 μm (PM10) in the capital, μg/m^3^ – 1980	N/A	N/A	20.4^2000^	N/A	N/A	34.7^2001^	35^2003^	N/A	N/A	N/A	52.7^2004^	36.5^1999^	30.9^2002^	N/A	N/A	N/A
Average annual concentration of particulate matter <10 μm (PM10) in the capital, μg/m^3^ – 2010	22.6^2009^	48.5	48.4	N/A	N/A	30.4^2007^	31.9	N/A	N/A	35.4	23.1	26.6	29.4^2009^	N/A	59.5	N/A
Average annual concentration of nitrogen dioxide (NO_2_) in capital, μg/m^3^ – 1980	N/A	27^2002^	39.7^2003^	N/A	N/A	50.8^1997^	53.2^1997^	N/A	N/A	N/A	40.2^2003^	34.3^1997^	31.6^2002^	N/A	N/A	N/A
Average annual concentration of nitrogen dioxide (NO_2_) in capital, μg/m^3^ – 2010	N/A	25.7	31.3	N/A	N/A	42.4^2008^	28.1	N/A	N/A	20.5^2011^	27.9	13.3	34.7	15^2009^	N/A	N/A
Average annual concentration of ozone (O_3_) in the capital, μg/m^3^ – 1980	N/A	68.4^2006^	4.4^1999^	N/A	N/A	75.8^1997^	69^1997^	N/A	N/A	N/A	65.4^2004^	72^1998^	66^1998^	N/A	N/A	N/A
Average annual concentration of ozone (O_3_) in the capital, μg/m^3^ – 2010	N/A	53.6	65.4	N/A	N/A	88.6^2008^	73.7^2009^	N/A	N/A	57.7	71.2^2011^	71.6	63.7	N/A	N/A	N/A
Total health expenditure, PPP$ per capita, WHO estimates – 1980	97.6^1995^	128.44^1955^	290.22^1995^	546.04^1995^	722.76^1995^	1263.1^1995^	656.74^1995^	445.18^1995^	115.12^1995^	183.44^1995^	259.86^1995^	503.8^1995^	969.4^1995^	421.4^1995^	174.12^1995^	246.56^1995^
Total health expenditure, PPP$ per capita, WHO estimates – 2010	481.9	833.74	1053.1	1461.7	2221.68	2584.6	1653.88	947.86	369.66	880.94	1183.44	2088.18	2366.4	772.02	1071.54	520.44

Containing epidemiological burden of COPD in the Balkans, while providing equitable and affordable medical care for patients will demand surmounting efforts from local communities. Economic consequences in terms of illness attributed lost productivity are huge and due to ongoing upward economic developments in the area likely to increase further. Current national capacities in SEE health care provision remain insufficient, not only in terms of professional staff but also in terms of specialized clinics and rehabilitation facilities, which are still scarce across the region (37). Through the course of past decades, historical network of facilities created to combat tuberculosis was seriously downsized due to successes of innovative vaccines and antibiotics. Another important issue is strong concentration of clinical physicians and nurses in urban cores, leaving rural areas underserved (38).

Far reaching potentially successful strategy to combat COPD in South Eastern Europe would have several distinct features. Such effort should be supranational and should contain key priorities defined within common EU policy on COPD (39). It would have to include peculiarities of local public health and clinical settings, which were already proven to affect resource use and outcomes of COPD medical care (40). Major measures assume prevention of smoking among youth and controlling environmental pollution primarily in large cities. Timely detection of illness by broadly targeted diagnostic screenings could allow more efficient treatment and preserving clinical evolution in its early stages. Evidence based allocation, favoring implementation of cost-effective diagnostic and treatment protocols would help to contain cost without significant adverse influence to the quality of care. Such a complex approach could allow larger portion of local communities to be taken care for, particularly among the poor and underserved citizens.

Although the quantification of the direct health care costs of COPD as well as indirect and intangible costs in these countries is very difficult, it is clear that pulmonary specialists across the South Eastern Europe region are challenged to increase their efforts to reduce the menace of smoking and to put in additional efforts in creation of new strategies aimed at early diagnostics. The estimation of total health care costs can therefore only be a first step in assessing the overall impact of COPD burden in South East Europe region. Further studies on the economic burden of COPD, including the perspective of mostly underestimated indirect and intangible costs within the region will be needed to prove and justify the prevention and early diagnostics efforts and development of new strategies of reduction of both financial and non-financial burden of disease. Many policy makers are starting to realize that a more robust evidence base is needed in order to make informed decisions on resource allocation. In light of current weaknesses of regional health financing, funding the quest for knowledge of the local cost drivers of key clinical conditions represents a valuable investment in the future of emerging markets (41).

COPD with its multimillion patient population in the SEE region should be regarded as one of the high-profile policy issues on the agenda of national health ministries and governmental agencies. Future of these patients remains particularly unpredictable among the small Western Balkan economies approaching EU membership.

## Conflict of Interest Statement

The author declares that the research was conducted in the absence of any commercial or financial relationships that could be construed as a potential conflict of interest.
